# Custom tissue engineered aneurysm models with varying neck size and height for early stage in vitro testing of flow diverters

**DOI:** 10.1007/s10856-020-06372-y

**Published:** 2020-03-14

**Authors:** Camille Villadolid, Brandon Puccini, Benjamin Dennis, Tessa Gunnin, Conor Hedigan, Kristen O’Halloran Cardinal

**Affiliations:** 1 Biomedical Engineering Department, Cal Poly, 1 Grand Ave, San Luis Obispo, CA 93407 USA; 2 Mechanical Engineering Department, Cal Poly, 1 Grand Ave, San Luis Obispo, CA 93407 USA

## Abstract

Endovascular techniques for treating cerebral aneurysms are rapidly advancing and require testing to optimize device configurations. The purpose of this work was to customize tissue-engineered aneurysm “blood vessel mimics” (aBVMs) for early stage in vitro assessment of vascular cell responses to flow diverters and other devices. Aneurysm scaffolds with varying neck size and height were created through solid modeling, mold fabrication, mandrel creation, and electrospinning. Scaffold dimensions and fiber morphology were characterized. aBVMs were created by depositing human smooth muscle and endothelial cells within scaffolds, and cultivating within perfusion bioreactors. These vessels were left untreated or used for flow diverter implantation. Cellular responses to flow diverters were evaluated at 3 days. Custom scaffolds were created with aneurysm neck diameters of 2.3, 3.5, and 5.5 mm and with aneurysm heights of 2, 5, and 8 mm. A set of scaffolds with varying neck size was used for aBVM creation, and dual-sodding of endothelial and smooth muscle cells resulted in consistent and confluent cellular linings. Flow diverters were successfully implanted in a subset of aBVMs, and initial cell coverage over devices was seen in the parent vessel at 3 days. Direct visualization of the device over the neck region was feasible, supporting the future use of these models for evaluating and comparing flow diverter healing. Tissue-engineered aneurysm models can be created with custom neck sizes and heights, and used to evaluate cellular responses to flow diverters and other endovascular devices.

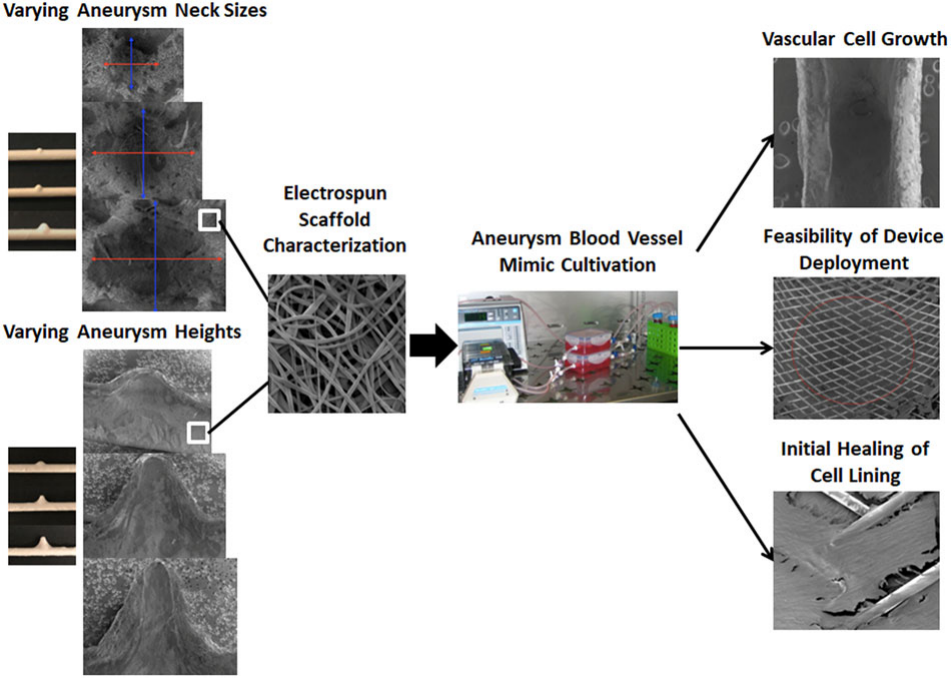

## Introduction

Tissue engineering can be used to create in vitro models for early assessment of drugs or devices [1–4]. Tissue-engineered “blood vessel mimics” (BVMs) [1–6] are vascular constructs composed of polymer scaffolds seeded and cultivated with human vascular cells [3]. BVMs have been used in their straight form for early stage device assessment, including for the evaluation of endovascular flow diverters [1], and they have the potential for more customized preclinical modeling of aneurysm disease states.

In a clinical setting, aneurysms occur when blood shear stresses cause a weakened spot in the arterial wall to bulge [7]. Over time, aneurysms will enlarge, and in the brain an untreated aneurysm can rupture and cause a subarachnoid hemorrhage [8]. One standard aneurysm treatment involves surgical intervention through clipping [9]; however, patients at a higher risk of surgical complications or those who have aneurysms in the posterior circulation may require a less invasive alternative treatment. Endovascular devices are delivered via an access point in the femoral artery. One endovascular option is to use detachable coils to densely pack the aneurysm sac [10], and stent-assisted coiling techniques have emerged from simple coiling to prevent coil migration out of the aneurysm [11]. It is also possible to use flow diverters, which are devices that limit flow into the aneurysm and promote healing across the neck [12].

The increasing prevalence of these endovascular devices necessitates proper preclinical models for evaluation. These models can be utilized at different stages, including traditional in vivo models, as well as earlier-stage bench and in vitro models. The most common in vivo aneurysm model is the rabbit elastase-induced aneurysm model [13–16]. In addition to studying aneurysm pathology, the rabbit elastase model has been used to evaluate different endovascular devices such as coils and flow diverters [17–20]. This model has also been modified to represent a larger variety of aneurysms found clinically by controlling height [21] and neck size [22].

Bench models most commonly include 3D silicone models replicated from clinical scans. These silicone models can incorporate a variety of aneurysm sizes and geometries, and have been used for hemodynamic flow studies, with and without devices [23–25] and for physician training [26].

In vitro models provide the possibility of evaluating vascular cell behavior in response to aneurysm geometries and device materials and coatings. The majority of previous in vitro work has been focused on studying cellular responses to hemodynamic conditions, as opposed to device testing. For example, Kaneko et al. [27] replicated a basilar tip aneurysm clinical scan and modeled it in PDMS coated with fibronectin to study flow differences on endothelial cell morphology. Similarly, Levitt et al. [28] created a hydrophilic plasma-treated PDMS model and quantified endothelial cell gene expression at different areas of the aneurysm geometry. Touroo et al. [5] dilated a portion of an ePTFE vascular graft to create a fusiform aneurysm-like geometry and pressure sodded stromal vascular fraction cells intraluminally. None of these in vitro models, however, deployed devices into their aneurysms.

Previous work by our laboratory developed aneurysm blood vessel mimics (aBVMs) with various aneurysm geometries to create in vitro models to test endovascular devices [1]. Customized molds of blister, saccular, and fusiform aneurysms were successfully modeled, and fibrous poly(lactic-co-glycolic) acid (PLGA) scaffolds were electrospun. This model demonstrated initial feasibility using immortalized mouse fibroblasts (3T3s) as a proof of concept cell type.

The goal of the current work was to explore whether aBVMs could be more specifically customized and utilized for device testing. The hypotheses were that electrospun aneurysm scaffold geometries can be customized specifically based on height and neck size, that these scaffolds can be used with human vascular cells to tissue engineer constructs, and that these constructs can be used for flow diverter implantation and testing. The current work was done specifically for testing flow diverters, where the aneurysm neck morphology is crucial. For other endovascular devices such as coils, the aneurysm height dimension is important as well for testing packing densities [29]. To perform this work, a comprehensive review of the rabbit elastase model was first performed to establish a specific range of desirable neck and height dimensions. Then, using this data, a series of custom electrospun aneurysm scaffolds were created with varied neck and height sizes. Aneurysm dimensions and scaffold morphology were characterized. Finally, the scaffolds with varying neck sizes were used in the in vitro aBVM application for evaluating flow diverters.

## Materials and methods

The present work included four distinct steps. The first step was to determine a range of appropriate and desirable dimensions for in vitro aneurysm models based on the in vivo rabbit elastase-induced aneurysm models. The second step was to create custom aneurysm scaffolds through solid modeling of selected dimensions and subsequent mold machining, mandrel fabrication, and electrospinning. This step included characterization of all resulting scaffold geometries. The third step was to demonstrate feasibility of the new geometries in aBVMs through construct cultivation using an endothelial cell and vascular smooth muscle cell dual-sodding technique. Lastly, the fourth and final step was to demonstrate the use of the dual-sodded aBVMs as a preclinical model for endovascular device testing by implanting and imaging flow diverters.

### Literature review to establish dimensions

A literature review was conducted using PubMed to determine the range of dimensions seen in aneurysm geometries from in vivo rabbit models. The following key words included in the search were: “cerebral aneurysm”, “cranial aneurysm”, “aneurysm geometry”, and “rabbit cranial aneurysm model”. Articles that were referenced in the studies found were also included in the review. The critical dimensions gathered from the studies included the diameter of the aneurysm neck and the aneurysm height. This information was used to select the neck sizes and heights used in the current studies.

### Solid model and mold creation

Once desired dimensions were determined, custom mandrel collectors for electrospinning were made by first creating solid models. Three different circular neck sizes were chosen, and molds were created using Solidworks (Dassault Systemes, 2017). Varied aneurysm heights with a constant neck diameter were also created in Solidworks. The 4 mm parent artery diameter remained constant throughout all iterations. Molds with the negative impression of the desired aneurysm geometries and dimensions were then computer numerical controlled (CNC) machined.

### Custom mandrel fabrication and scaffold creation

Custom mandrel fabrication and electrospun scaffold creation was performed similar to the methods described previously by Shen et al. [1]. Once the molds were machined, a small-diameter stainless steel mandrel was placed within the mold in the groove of the parent artery. Water-soluble wax (Freeman Sol-U-Carv Wax, 263–1051 or Westech Wax Products Hydrosol 3030H Soluble Wax, 1003980) was melted and injected into the mold to create a customized aneurysm mandrel to place into the electrospinner. Different mandrels were created for each desired geometry. Then, plastic wrap was heat shrunk onto the mandrels.

A 15% weight solution of poly(D,L-lactide-*co*-glycolide) (PLGA, Sigma, p1941) dissolved in chloroform (AcroSeal, 326820010) was prepared for electrospinning. After mixing, the solution was loaded into a syringe pump and electrospun onto the customized aneurysm mandrels [1]. Electrospinning parameters included an applied voltage at −12 kV, a gap distance of 10 inches, and a flow rate of 4.5 ml/h. After electrospinning, each polymer-covered mandrel was cut in half radially to produce two aneurysm scaffolds. The scaffolds were then cut off the mandrel and characterized or used in an aBVM model.

Scaffolds that were used in the aBVM model underwent additional steps during removal from the wax mandrel. The wax was first softened by placement of the entire scaffold and mandrel in an oven to allow the scaffold to slide off the metal mandrel. Then, the residual wax was dissolved in Dulbecco’s modified Eagle medium (DMEM). After 40 min of dissolving, the plastic wrap was pulled out and the scaffold was intact and ready for subsequent use in aBVM cultivation.

### Aneurysm scaffold characterization

Internal measurements were made for the critical dimension of each aneurysm scaffold to confirm that scaffolds reflected the designs and molds appropriately. This meant that neck diameters were characterized for the varying neck scaffolds, and height was characterized for the varying height scaffolds. These internal dimensions were measured by cutting scaffolds open, taking SEM images, and measuring desired dimensions with ImageJ. When preparing a scaffold for internal imaging, the parent artery on either side of the aneurysm was removed. To visualize the aneurysm neck, the bottom half of the parent vessel was cut out to expose the entire luminal neck. To visualize the aneurysm height, the vessel was cut longitudinally to yield two symmetrical side views of the inside of the aneurysm dome. Samples were then sputter coated with gold for 70 s (Denton Vacuum, Desk IV), and prepared for SEM imaging by mounting on aluminum stubs (Ted Pella, Inc, 16144) with double-sided carbon tape (Ted Pella, Inc, 16084-2). Conductive copper tape (Ted Pella, Inc, 16072) was used with some samples. Images of the luminal sides of scaffolds were taken with a scanning electron microscope (JEOL, JSM-6390) in secondary electron imaging (SEI) mode. The voltage was 1.0 kv or 1.5 kv and working distance ranged from 10 to 46 mm, depending on the sample size and desired magnification. The neck diameters both parallel and perpendicular to the parent vessel were measured for the varying neck scaffolds, and the heights were measured for the varying height scaffolds. These measurements were made in ImageJ using the “Straight Line” tool. Each measurement was taken three times, and the average was recorded.

SEM images were also taken at higher magnifications in order to confirm consistent scaffold microstructure. The fiber diameter was measured in nine different portions of each image, using ImageJ along with a grid overlay for selection of fibers to measure, and the average was calculated with a standard deviation.

### aBVM cultivation

To demonstrate feasibility of using the new scaffold geometries as in vitro aBVMs, and to apply our vascular cell dual-sodding approach to aBVMs, aneurysm scaffolds were prepared and cultivated as described previously for straight BVMs by Herting et al. [3]. Briefly, human umbilical vein endothelial cells (HUVECs, Lonza, C2519A) and human umbilical artery smooth muscle cells (HUASMCs, Cell Applications Inc., 252-05n) were cultured at 37 °C and 5% CO_2_ with smooth muscle cell medium (Cell Applications Inc., 310–470) and endothelial cell medium (Clonetics EBM-2, Lonza CC-3156). Cells were split at 1:2 or 1:3 ratios until a sufficient cell population was obtained for cell sodding. Smooth muscle cells were used by passage 8, and endothelial cells were used by passage 7.

Two aneurysm scaffolds of each neck size (2.3, 3.5, and 5.5 mm in diameter) were electrospun and prepared for this study, as described above. Scaffolds were cut into 4.0 cm-long sections for aBVM cultivation. Barbed fittings were placed on each end of the sections, and scaffolds were sterilized with 70% ethanol. Scaffolds were then flushed with DPBS followed by M199 enriched with fetal bovine serum, P/S, and Fungizone before being mounted into individual sterile bioreactor chambers. The bioreactor chambers were filled with M199 and fetal bovine serum plus L-glutamine, P/S, Fungizone, and HEPES buffer, and were connected to a media reservoir with gas permeable tubing. The closed-loop system was run on a peristaltic pump. Transmural flow, which allowed the protein-rich media to penetrate through the pores of the scaffold, was administered for 12 h to “condition” the scaffold in preparation for cell deposition.

Following scaffold conditioning, a dual-sodding technique was performed to deposit cells within the aneurysm scaffolds. First, HUASMCs were harvested and pressure sodded into the luminal wall of each scaffold, as previously described [3]. Following a 3-hour static period, HUVECs were harvested and deposited into the construct. An additional 2-h static period followed. The protein-rich conditioning media was then switched to a “growth medium,” which was made with a 3:1 ratio of EC and SMC media, respectively. Transluminal flow was applied with the peristaltic pump beginning at 15 rpm and gradually increased to 90 rpm over the course of 9 h. After 36 h of cultivation, one construct of each geometry was utilized for device deployment, while the other construct of each geometry was left untreated and maintained in cultivation as a control.

### Device deployment

One aBVM of each neck diameter was randomly chosen for device deployment to show feasibility of device testing using the different neck size iterations of the aBVM model. Thirty-six hours after sodding, designated aBVM systems were removed from the pump and incubator and brought into the biological safety cabinet where a 4.5 × 15 mm self-expanding, CoCr and Pt braided flow diverter (supplied by Stryker Neurovascular) was deployed aseptically into each designated aBVM. Following deployment, each bioreactor remained static for 2 h and was then reconnected to the peristaltic pump for continuing dynamic cultivation. After three days, all constructs were harvested. The 3-day time point was selected in order to evaluate the ability of vessels to withstand device deployment and support initial cell interaction.

### aBVM harvest and analysis

Three days after device deployments, all six constructs were removed from bioreactors and fixed in 2.5% glutaraldehyde. After fixation, vessels were dehydrated in a series of ethanol solutions gradually increasing in concentration. Then, they were cut longitudinally so that one half contained the entire aneurysm dome and the other half was only the parent vessel. The luminal surface of each sample was sputter coated for 60 s (Denton Vacuum, Desk IV) and then imaged using a scanning electron microscope (JEOL, JSM-6390) to visualize the cell lining.

## Results

### Literature review to establish dimensions

Results from the literature review revealed a range of aneurysm geometries and dimensions that exist within the rabbit elastase model. The minimum neck diameter noted was 2.3 mm from Ding et al. [22], whereas the largest neck diameter was found to be around 5.5 mm. Aneurysm heights were much more variable; some of the heights were less than 2 mm, and one study achieved heights of more than 16 mm [21], though most heights measured around 6–8 mm tall. The compilation of this data (Table 1) suggested that in vitro models should ideally be able to incorporate neck sizes ranging from 2.3 to 5.5 mm in diameter, and heights ranging from 2 to 8 mm in order to replicate standard rabbit aneurysm sizes.

**Table 1:** Summary of aneurysm dimensions seen in the rabbit elastase model

Author	Treatment	Neck diam. (mm)	Dome diam. (mm)	Height (mm)
Kainth et al.	Single	2.6–2.9	2.9–3.4	4.8–6.1
	Double	3.2–3.7	2.5–3.1	1.7–2.4
Brinjikji et al.	–	–	4.5	7.5
Ding et al.	AVF + Elastase	3.5–5.5	3.4–6.4	8.6–16.8
	Control	2.5–4.3	2.9–4.3	6.4–9.5
Altes et al.	–	–	3–6.5	5–10
Li et al.	–	2.81–5.48	2.81–6.73	2.67–9.23
Ding et al.	Low Balloon	3.4 ± 1.2	3.8 ± 1.0	8.0 ± 1.7
	High Balloon	2.3 ± 0.9	3.3 ± 0.9	7.5 ± 2.2
King et al.	PED w/DAPT	3.8 ± 0.7	3.3 ± 0.8	6.3 ± 1.2
	PED w/oDAPT	4.1 ± 1.3	3.8 ± 0.9	7.7 ± 2.2
	sPED w/DAPT	4.3 ± 1.8	3.4 ± 1.0	7.7 ± 2.3
	sPED w/oDAPT	3.9 ± 1.0	3.8 ± 0.9	7.6 ± 2.5
Marosfoi et al.	48-wire FD	5.3 ± 1.9	–	6.9 ± 1.7
	72-wire FD	4.7 ± 1.3	–	7.1 ± 1.6
Li et al.	–	4.57 ± 0.19	5.14 ± 0.24	7.21 ± 0.48

### Solid model, mold, mandrel, and scaffold creation for varying neck size

**Fig. 1:**
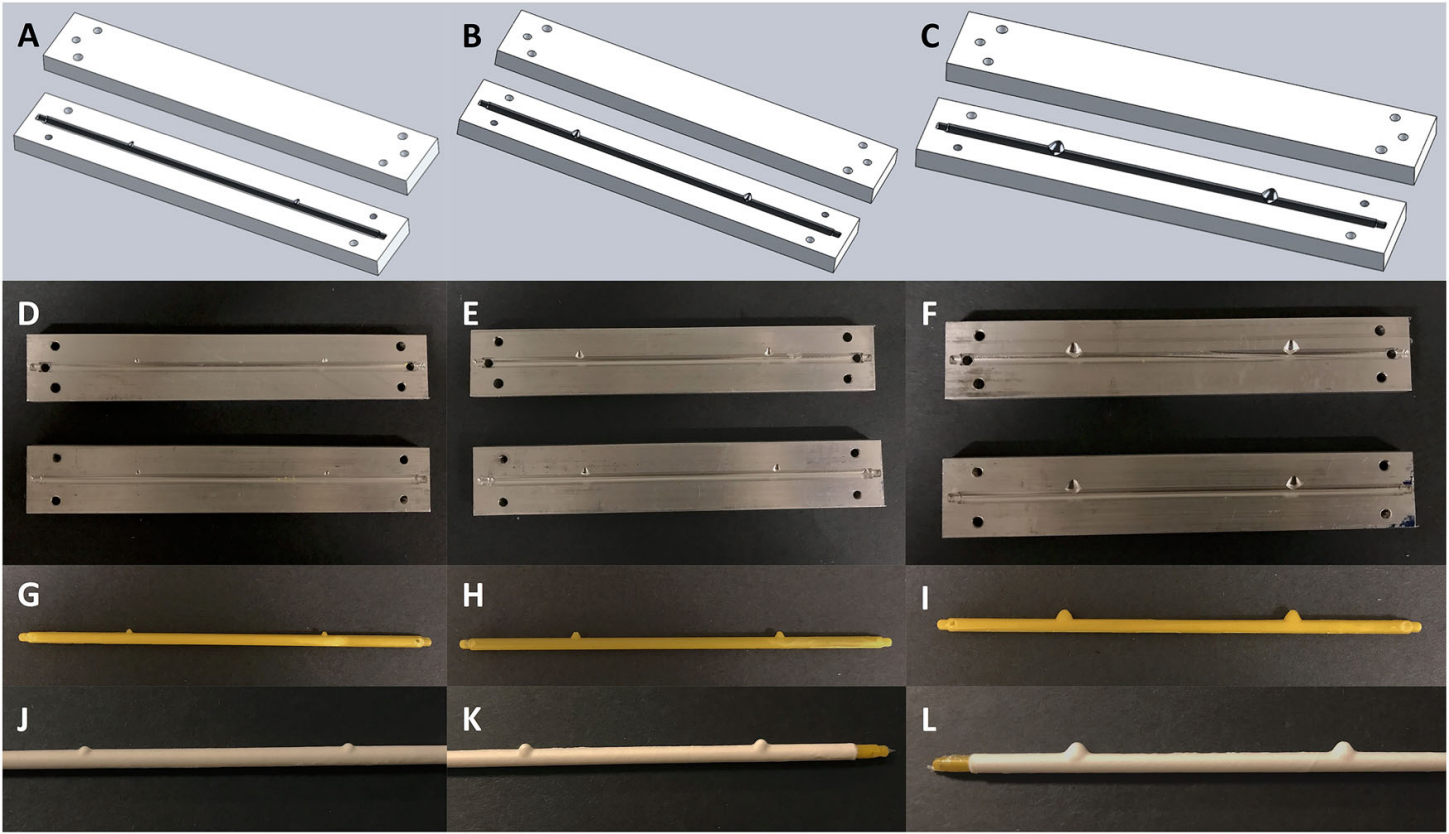
Creation of custom electrospun aneurysm scaffolds with varying neck sizes. Aneurysm neck diameters of 2.3 mm (left column), 3.5 mm (middle column), and 5.5 mm (right column) were designed using Solidworks (**a**–**c**) and machined into molds (**d**–**f**). Then, casts were created using the molds from water-soluble wax (**g**–**i**), and PLGA scaffolds were electrospun over the custom mandrels (**j**–**l**). Each parent vessel had a diameter measuring 4 mm

Custom scaffolds with neck diameters ranging from 2.3 to 5.5 mm were successfully created. Aneurysm models were cone shaped to allow for electrospun fibers to collect tightly at the neck, as illustrated in Fig. 1a–c. Molds were then machined and custom mandrels were created using the wax injection methods described, as seen in Fig. 1d–i. This process was consistent regardless of neck size. Following mold and mandrel creation, the standard electrospinning process was successful on all three geometries, yielding polymer scaffolds with varying neck sizes, as illustrated in Fig. 1j–l.

### Aneurysm scaffold characterization for varying neck size

**Fig. 2:**
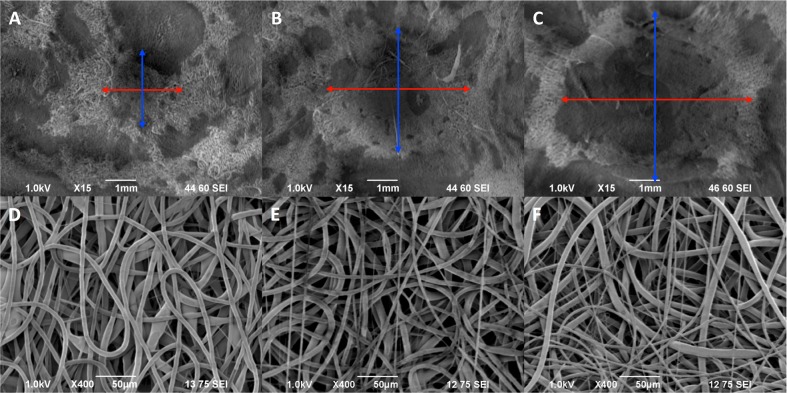
Characterization of aneurysm scaffolds with varying neck diameters of 2.3 mm (left column), 3.5 mm (middle column), and 5.5 mm (right column). The inner lumens at the aneurysm necks were SEM imaged at ×15 magnification to visualize neck dimensions (**a**–**c**). Red arrows indicate the parallel neck diameters, and blue arrows indicate the perpendicular neck diameters. Flow direction would be oriented horizontally. Representative SEM images at ×400 magnification illustrated consistent fiber morphology and diameter for each scaffold (**d**–**f**)

Following completion of scaffolds with varying neck sizes, SEM characterization was performed to determine internal neck dimensions, as illustrated in Fig. 2a–c. For the 2.3 mm neck scaffold, the actual neck dimensions were measured to be 2.09 × 2.28 mm (where the neck diameter parallel to the flow direction is listed first). For the 3.5 mm neck scaffold, the actual dimensions were measured to be 3.58 × 3.58 mm. For the 5.5 mm neck scaffold, the actual neck dimensions were measured to be 5.51 × 5.67 mm. Average fiber diameters, as illustrated in Fig. 2d–f, were measured to be 5.91 μm ± 1.46 μm for the 2.3 mm neck scaffolds, 5.28 μm ± 2.78 μm for the 3.5 mm neck scaffolds, and 5.27 μm ± 2.26 μm for 5.5 mm neck scaffolds.

### Solid model, mold, mandrel, and scaffold creation for varying height

**Fig. 3:**
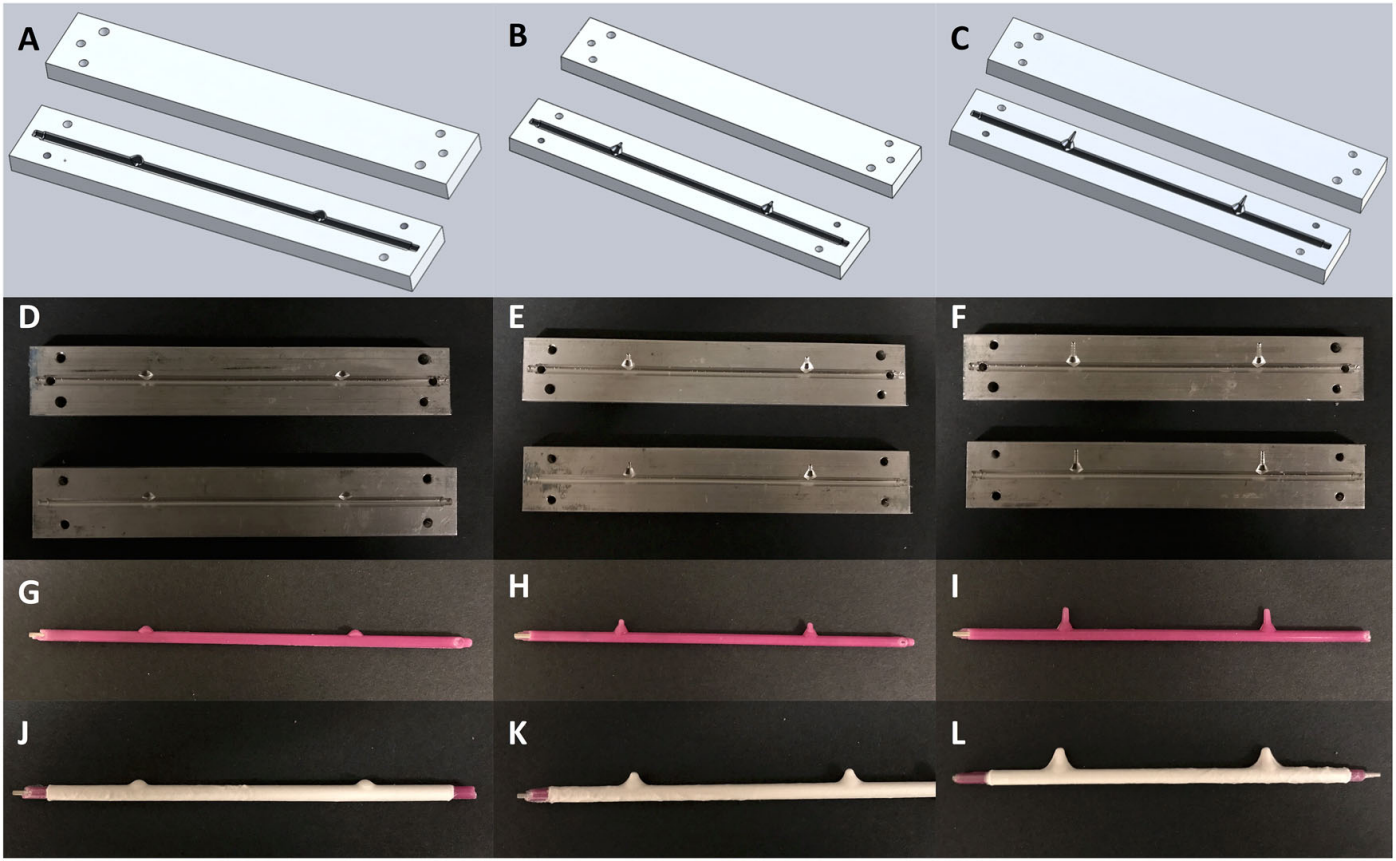
Creation of custom electrospun aneurysm scaffolds with varying heights. Aneurysm heights of 2 mm (left column), 5 mm (middle column), and 8 mm (right column) were designed using Solidworks (**a**–**c**) and machined (**d**–**f**). Then, casts were created using the molds from water-soluble wax (**g**–**i**), and PLGA scaffolds were electrospun over the custom mandrels (**j**–**l**). Each parent vessel had a diameter measuring 4 mm

Custom scaffolds with various aneurysm heights ranging from 2 to 8 mm were successfully created. The 5 and 8 mm height models included an additional cone shaped extrusion so that the desired height was achieved, as illustrated in Fig. 3a–c. Molds were machined and custom mandrels were created using the wax injection methods described, as seen in Fig. 3d–i. Following mold and mandrel creation, the standard electrospinning process was successful on all three geometries, yielding polymer scaffolds with varying heights, as illustrated in Fig. 3j–l.

### Aneurysm scaffold characterization for varying height

**Fig. 4:**
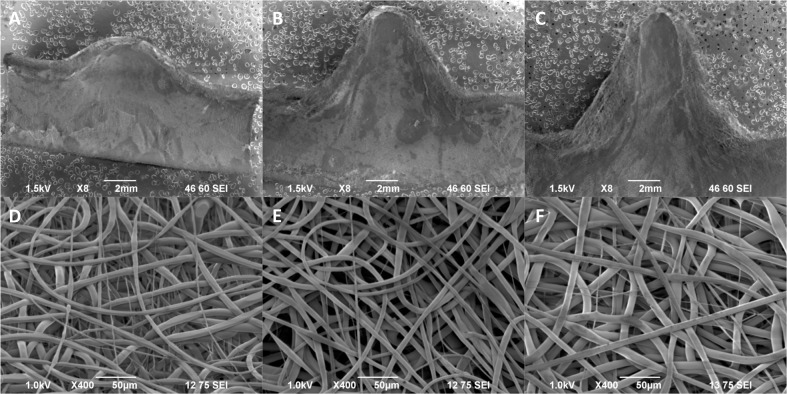
Characterization of aneurysm scaffolds with varying heights of 2 mm (left column), 5 mm (middle column), and 8 mm (right column). A symmetrical half of each scaffold was SEM imaged at ×8 magnification (**a**–**c**). Flow direction would be oriented horizontally. Representative SEM images at ×400 magnification illustrated consistent fiber morphology and diameter for each scaffold (**d**–**f**)

Following completion of scaffolds with varying height, SEM characterization was performed to determine the internal height dimension, as illustrated in Fig. 4a–c. For the 2 mm height scaffold, the actual height was 2.2 mm. For the 5 mm scaffold, the actual height was 5.61 mm. For the 8 mm height scaffold, the actual height was 8.35 mm. Average fiber diameters, as illustrated in Fig. 4d–f, were measured to be 5.09 μm ± 2.11 μm for the 2 mm height scaffolds, 5.03 μm ± 1.32 μm for the 5 mm height scaffolds, and 6.61 μm ± 2.29 μm for the 8 mm height scaffolds. For the 8 mm height scaffolds, a thin layer of uniaxial aligned fibers was observed at the base of the neck before webbing outwards on either side of the aneurysm. Overall, scaffold microstructure was similar between geometries, indicating that these different geometries can be created using the standard electrospinning process.

### aBVM cultivation

**Fig. 5:**
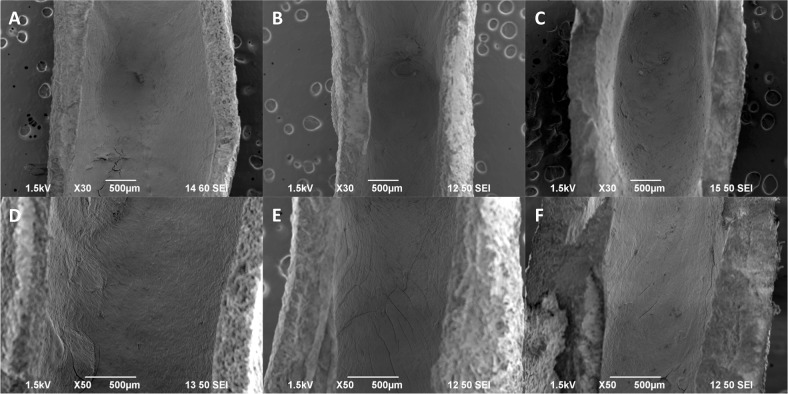
aBVM cultivation of aneurysm scaffolds with varying neck diameters of 2.3 mm (left column), 3.5 mm (middle column), and 5.5 mm (right column) using a dual-sodding technique for cell deposition. Flow direction was oriented vertically for all vessels. Aneurysms were imaged at ×30 magnification (**a**–**c**) and exhibited consistent and confluent cell linings, and parent artery images at ×50 magnification (**d**–**f**) also illustrated confluent cell linings in each vessel

Following creation and characterization of custom neck sizes and heights for aneurysm BVM scaffolds, the next step was to apply our standard dual-sodding approach to new geometries to determine if EC and SMC deposition and vessel cultivation was feasible. The subset of scaffolds with neck variations were chosen because of their relevance to flow diverter treatments, which were tested for feasibility in straight BVMs previously in our lab [1]. The dual-sodding approach was successfully performed, and a consistent cell lining was observed in all areas of the scaffold within the aneurysm (Fig. 5a–c) and in the parent vessel lumen (Fig. 5d–f). The cell lining was consistent throughout all geometries, indicating that the dual-sodding approach can be used in different aneurysm scaffold versions.

### Flow diverter deployment and assessment in aBVMs with varying neck size

**Fig. 6:**
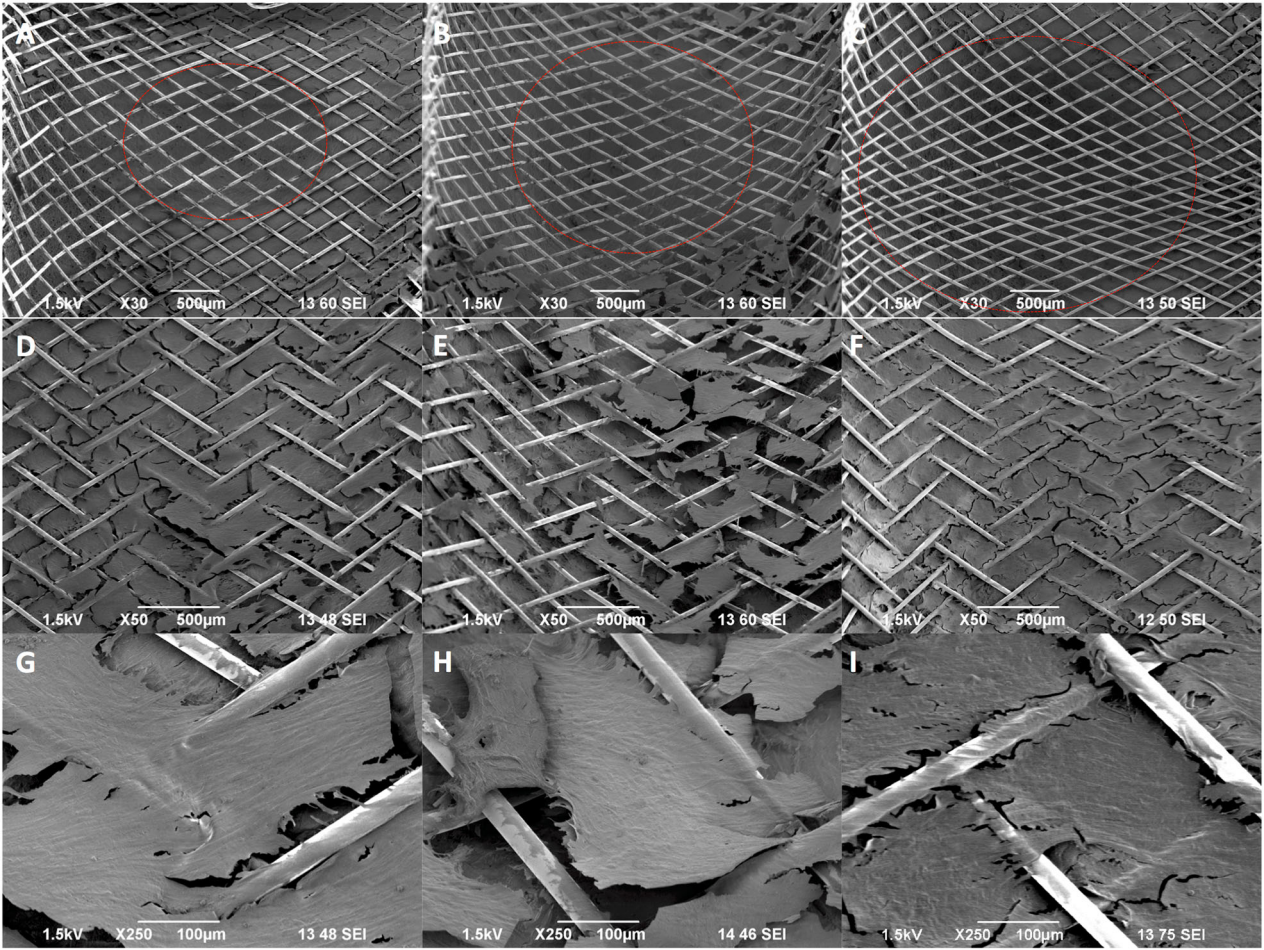
Evaluation of flow diverters in aBVMs with varying neck diameters measuring 2.3 mm (left column), 3.5 mm (middle column), and 5.5 mm (right column) after 3 days. SEM images of the implanted flow diverters over the aneurysms (outlined with red dotted circles) were taken at ×30 magnification (**a**–**c**). SEM images of the implanted flow diverters over the parent vessel were taken at ×50 magnification (**d**–**f**) and ×250 magnification (**g**–**i**). Flow direction was oriented vertically. Images illustrated partial cell coverage over portions of the flow diverter struts at the parent vessel region

The final step of this work involved deploying flow diverters into aBVMs with varying neck sizes. Scaffolds with neck diameters of 2.3, 3.5, and 5.5 mm were electrospun, and ECs and SMCs were dual-sodded and cultivated in a perfusion bioreactor to create the aBVMs. Flow diverters were then successfully implanted in the aneurysm vessels. These aBVMs with flow diverters were cultivated for three days before harvest and analysis. Aneurysm neck regions were able to be clearly visualized by SEM (Fig. 6a–c), and initial cell coverage was observed over devices in parent vessel regions (Fig. 6d–i), suggesting that these vessels can support and withstand flow diverter deployment and that healing in the parent vessel and at the neck region can be assessed by visualization of cell growth over device struts.

## Discussion

The goal of this work was to customize and utilize tissue-engineered aneurysm models for early stage, in vitro testing of endovascular devices. The hypotheses were that electrospun aneurysm scaffolds could be customized based on height and neck size and used with human vascular cells to tissue engineer constructs, and that these constructs could be used for flow diverter implantation and testing. Results illustrated that custom aneurysm scaffolds with varying neck size and height were successfully modeled, molded, and electrospun, which increases the model’s compatibility with different types of hemorrhagic stroke devices. Through visualization of scaffold lumens using SEM imaging, distinct neck diameters and aneurysm heights were achieved, and scaffold microstructure and fiber diameter were relatively consistent throughout all geometries, with randomly dispersed, micron-sized fibers similar to those previously documented with our electrospinning process [1]. Human endothelial cells and smooth muscle cells were dual-sodded onto the scaffolds with different neck sizes to show feasibility of these geometries for use as aneurysm blood vessel mimics (aBVMs). All constructs had consistent cell linings throughout all parts of the scaffold after cultivation in a perfusion bioreactor, supporting the success of vessel creation. Flow diverter implantation was performed in the varying neck size geometries. Results after three days of cultivation showed that all three different geometries were able to facilitate and withstand deployment, with initial cell coverage seen in parent vessel regions. In addition, aneurysm vessels were cut, processed, and imaged to allow direct visualization of the device over the neck region, which supports the future use of this model for evaluating healing over the neck region.

Based on the review of literature from the rabbit elastase model, it was determined that neck sizes ranging from 2.3 to 5.5 mm would be desirable in our model, so diameters of 2.3, 3.5, and 5.5 mm were modeled and created. It was also determined that the heights of 2 to 8 mm would be desirable, so scaffolds with 2, 5, and 8 mm heights were modeled and created. Using the methods described in this work, other geometries or dimensions could also be created, although larger neck sizes would require either a larger parent vessel diameter or a more elliptical neck geometry. In addition, aneurysm domes were designed to be slightly cone-shaped for optimal fiber collection during electrospinning, and more saccular geometries would require a different fiber deposition approach; however, for flow diverters and for the current work, the neck diameter itself was deemed more important than the geometry above it.

Previous work from our laboratory has shown cell deposition within aneurysm scaffolds [1], but this was the first documented use of the electrospun aneurysm geometries with our dual-sodding approach of applying human smooth muscle cells and endothelial cells. Having both cell types in the aBVM model can facilitate future evaluations of specific cell responses to various endovascular device configurations; however, immunostaining or other phenotype-specific assessment methods would be required to make definitive conclusions about the different cell types. For the current studies, SEM images demonstrating smooth, consistent, and confluent cell linings throughout the entirety of each vessel were deemed successful.

Although the current work illustrated development of aBVMs in a range of sizes and geometries for testing different types of endovascular devices, flow diverters were specifically selected for utilization in these studies; therefore, aBVMs with varying neck sizes were created for device implantation since the neck dimension is the most crucial consideration for these devices [30–32]. Six aBVMs with varying neck sizes were created, and three flow diverters were implanted in different geometries. Among each of the geometries, device deployment in aBVMs was straightforward and simple, as it was performed through direct visualization in a biological safety cabinet, with no fluoroscopy required. Following successful implantation, treated vessels and untreated controls were cultivated for 3 days. The purpose of this time point was to assess utility of the model for deployment, the ability to image and evaluate the neck, and any initial cell responses in the parent vessel. Results showed that the model withstood flow diverter deployment, and that cell coverage over device struts had begun to take place within the parent vessel. This time point was too short to expect healing over the neck region [33, 34]. In addition, dehydrating, cutting, and imaging of vessels were successful, resulting in direct visualization of the neck region. This supports the future use of this model for studying cell responses to flow diverter implantation in varying neck sizes and comparing healing using different device configurations over the neck region.

In comparison to other preclinical models, the aBVMs presented here have some advantages and some limitations. Other early stage models typically include benchtop models, which are often made of silicone or PDMS. Silicone models have shown great utility for hemodynamic studies, early device investigations, and physician training [26], although the biologic aspect of vascular cells is often missing. In a few recent publications, which incorporate endothelial cells into these benchtop models, devices were not deployed or assessed [27, 28]. Additionally, as compared with silicone, electrospun scaffolds have the advantage of a fibrous microstructure to enhance cell adhesion and vessel development. With regard to the geometries, many benchtop models are created based on a clinical scan [35–37], whereas ours were based on specifications that we determined. There may be advantages to both approaches.

When compared to the standard rabbit elastase model or other in vivo aneurysm models, aBVMs lack the complexity and dynamic physiologic environment of a living organism, and it is unlikely that our aBVM or other in vitro models will ever completely take the place of in vivo testing; however, the work here demonstrates that geometries and dimensions can be customized to be similar, with perhaps more control and reproducibility in the in vitro setting. In addition, aBVMs are less costly than animal studies and require less personnel and infrastructure, making them efficient to set up and use for early stage assessments and as a precursor to animal models. This addition to the device testing pipeline could reveal important insights to aid in planning future studies, ultimately reducing time and costs associated with subsequent in vivo studies. aBVMs can also incorporate human cells.

Although the aBVMs presented and utilized in the current work have important advantages and numerous potential uses, there are several limitations. With regard to the scaffolds, many geometries are possible, but not every combination was feasible. Height, for example, was limited by the size of the neck so that the Solidworks dome feature could generate cone-shaped aneurysms. As a result, smaller neck sizes could not accommodate a large range of heights, at least based on the Solidworks features we utilized. With regard to the environment, pumps and bioreactor systems can be tailored to incorporate physiologic flows and pressures, but the current work utilized a low, steady flow. This effectively provided nutrient and waste transport as well as a small amount of shear stress, which was sufficient for the current work; however, modification of the bioreactor systems would be necessary to recreate the appropriate hemodynamic environment. Based on the well-documented benchtop silicone models, this is certainly feasible [23–25, 38, 39]. Finally, with regard to the biologic aspect, no circulating cells are present in this model, so the cellular response that is assessed comes purely from the parent vessel.

Numerous future studies can be derived from the current work. Most immediately, experiments could be designed to use the varying neck aBVMs to study healing at longer time points and to compare healing between different device configurations, materials, or coatings. It would also be possible to use the varying height aBVMs to evaluate utility for coils, foams, or other devices, and to explore the incorporation of blood or circulating cells to increase relevance for those devices. Future work should also implement immunostaining to identify and understand the roles of the different cell types in response to the various devices.

## Conclusion

In conclusion, the work presented here made three important contributions towards the larger goal of creating and using tissue-engineered aneurysm models for early stage in vitro testing of endovascular devices. First, methods were established to customize and control aneurysm neck and height dimensions, which will allow for different types of devices to be properly evaluated. Second, a dual-sodding protocol using human endothelial and smooth muscle cells was successfully applied to custom scaffolds. Finally, flow diverters were deployed within aBVMs and demonstrated initial healing in the parent vessel region as well as the ability to directly image and assess the aneurysm neck region. These findings will allow early stage assessments and comparisons of new endovascular device coatings and materials.
